# Unraveling the Autism Spectrum Heterogeneity: Insights From ABIDE I Database Using Data/Model‐Driven Permutation Testing Approaches

**DOI:** 10.1002/brb3.71121

**Published:** 2026-01-31

**Authors:** Francisco José Alcaide, Ignacio Alvarez Illan, Javier Ramírez, Juan Manuel Gorriz

**Affiliations:** ^1^ DaSCI Institute University of Granada Granada Spain; ^2^ ibs.Granada Granada Spain

**Keywords:** linear support vector machines, magnetic resonance imaging, permutation tests, statistical learning theory, statistical parametric mapping, upper bounding

## Abstract

**Purpose:**

Autism spectrum condition (ASC) is a neurodevelopmental condition characterized by impairments in communication, social interaction, and restricted or repetitive behaviors. Extensive research has aimed to identify structural brain distinctions between individuals with ASC and neurotypical individuals using neuroimaging techniques. However, limited attention has been given to evaluating how variations in image acquisition protocols across different centers influence these observed differences.

**Method:**

This analysis focuses on structural magnetic resonance imaging (sMRI) data from the Autism Brain Imaging Data Exchange I (ABIDE I) database, considering both subjects' condition and individual centers to identify disparities between ASC and control groups. Statistical analysis, employing permutation tests, utilizes two distinct statistical mapping methods: statistical agnostic mapping (SAM) and statistical parametric mapping (SPM).

**Finding:**

Results from the SAM mapping method show greater consistency with existing literature. However, no statistically significant differences were found in any brain region. This outcome is attributed to factors such as limited sample sizes within certain centers, noise effects, and the challenges posed by multi‐center databases in a heterogeneous condition such as autism.

**Conclusion:**

The study indicates limitations in using the ABIDE I database to detect structural differences in the brain between neurotypical individuals and those diagnosed with ASC. Multi‐center variability and sample size constraints significantly affect the reliability of findings in structural neuroimaging studies of autism.

## Graphical abstract

Highlights of the Manuscript:

- Investigates the influence of cofactors in differentiating Autism Spectrum Disorder (ASD) from neurotypical individuals using the ABIDE database.
- Utilizes statistical analysis and permutation testing approaches to evaluate disparities between ASD and control groups.
- Reveals the absence of statistically significant differences in gray matter tissues across brain regions.

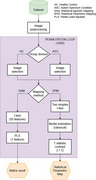

**Abbreviations**
ABIDE I: Autism Brain Imaging Data Exchange I
TD: typically developing
ASC: Autism Spectrum Condition
HC: healthy control
MRI: magnetic resonance images
SAM: Statistical Agnostic Mapping
SPM: Statistical Parametric Mapping
VBM: voxel‐based morphometry
TBV: total brain volume
GM: grey matter
WM: white matter
SBM: surface‐based morphometry
SVM: support vector machines
CAD: computer‐aided diagnosis
CNN: Convolutional Neural Network
fMRI: functional magnetic resonance imaging
sMRI: structural magnetic resonance imaging
DNN: Deep Neural Network
GCN: Graph Convolutional Network
AAL: Automated Anatomical Labeling
ROI: regions of interest
CV: cross‐validation
FWE: Family‐wise Error
PLS: Partial Least Squares Regression
GLM: General Linear Model
ReML: restricted maximun likelihood
FES: Feature Extraction and Selection
ABIDE: Autism Brain Imaging Data Exchange
ML: Machine Learning

## 1 Introduction

Autism spectrum condition (ASC), also referred to as autism, is a neurodevelopmental disorder with significant implications for communication, social interaction, and behavioral patterns (Lombardo et al. 2019; Masi et al. 2017; Szatmari 1999). The condition exhibits a complex nature, encompassing a broad spectrum of symptoms and severity levels, including significant gender imbalance (Górriz et al. 2019), thereby posing challenges in terms of diagnosis and treatment (Hodges et al. 2020; Rutherford et al. 2016). Nonetheless, our understanding of the inherent cerebral distinctions between neurotypical individuals and those with ASC remains limited. Efforts have been made over the years to address this knowledge gap (Ecker 2017), prompting researchers to employ neuroimaging methodologies for investigating the structural and functional aspects of the brain in individuals with ASC (Rafiee et al. 2022; Verhoeven et al. 2010). Although promising results have emerged from studies focusing on brain connectivity through functional MRI (fMRI) in ASC patients(Rane et al. 2015; Jones et al. 2016), this approach falls outside the scope of the present study.

Research has shown differences in brain structure between individuals with autism and neurotypical individuals(Ecker 2017), including overgrowth of the frontal cortex (Donovan and Basson 2016), reduced grey matter volume in specific brain regions (Toal et al. 2009; Gori et al. 2015; Segovia et al. 2014) or atypical regional gray matter volumetric changes (Wang et al. 2021), increased total brain and gray matter volumes (Riddle et al. 2016), and structural brain differences in areas implicated in social cognition and communication (K. Hyde et al. 2009). Longitudinal studies have also revealed age‐related reductions in total brain volume (McAlonan et al. 2005, 2008) and cortical thickness in individuals with autism (Hardan et al. 2009), and age‐dependent regional brain volume differences(Hsiang‐Yuan Lin et al. 2015; Li et al. 2019; Lincoln et al. 1995; Huemer et al. 2016). Meta‐analysis further confirmed consistent structural abnormalities in the ASC brain(T. Nickl‐Jockschat et al. 2012), as well as the ENIGMA consortium has confirmed altered structural brain asymmetry in the biggest ASC database to date(Postema et al. 2019). These findings collectively suggest that individuals with autism have distinct brain structural differences compared to neurotypical individuals. However, it is frequent in the ASC literature to report inconsistencies between findings (Katuwal et al. 2016; Zhang et al. 2018).

### 1.1 ABIDE Database and the Use of Machine Learning

Comprehensive and extensive datasets are required to further our understanding of the underlying brain mechanisms associated with autism and the intricate and heterogeneous nature of the disorder. To address this need, the Autism Brain Imaging Data Exchange I (ABIDE I) initiative was established(Di Martino et al. 2014). ABIDE I aims to collect functional and structural brain imaging data from multiple research laboratories worldwide, providing a valuable resource for advancing our knowledge of ASC.

Machine learning (ML) methods hold promise for extracting meaningful insights from limited neuroimaging samples, as often encountered in ASC research. Classical ML algorithms, such as SVM(Ecker et al. 2010) or Random Forest(Ecker et al. 2010) have been extensively used to identify distinctive features in neuroimages, aiding in the differentiation between subjects with accuracies reaching 90%.

However, significant heterogeneity exists among various analyses in autism research. This variability stems from factors such as the limited number of available ASC patients and the use of different datasets. When considering structural data of the ABIDE dataset, classical ML techniques have reported low differential capabilities (<60%) (Katuwal et al. (2015)) and recent advances using deep learning have increased their estimated generalization performance ranging from around 70% (Gao et al. 2021; Rakić et al. 2020) to around 90%(Kong et al. 2019). Despite this, there has been limited evaluation of how heterogeneity of ABIDE database may influence these discrepancies.

This study investigates the structural MRI data from the multicenter and International ABIDE I database (Di Martino et al. 2014) to explore its utility in characterizing ASC. Although some studies report high classification accuracy in distinguishing ASC from healthy control (HC) using ML techniques (Kong et al. 2019), there is still no clear consensus on specific structural differences or consistently affected brain regions in ASC, highlighting the need for approaches that provide both statistical rigor and anatomical interpretability. While deep learning methods excel at classification, they typically require very large datasets, can be prone to overfitting, and often lacks direct anatomical interpretability. In contrast, classical statistical mapping approaches, such as statistical parametric mapping (SPM) (Friston et al. 1994), and region of interest (ROI)‐based methods like statistical agnostic mapping (SAM) (Gorriz et al. 2021), provide voxel‐ and region‐level inference, control of false positives (FPs) through permutation testing, and results that are anatomically meaningful.

In this study, we hypothesize that combining SPM and SAM with extensive permutation testing can identify structural brain regions that significantly differ between ASC and HC. By comparing the outcomes of SPM and SAM, we assess their agreement: concordant findings provide strong evidence for true structural differences, whereas discrepancies highlight regions that warrant further investigation.

To disentangle true neurobiological differences in ASC from variability arising from other factors, we designed two complementary experiments. First, we analyzed the ABIDE dataset on a center‐by‐center basis, investigating whether differences identified in one center could be replicated across others. Second, we performed condition‐based comparisons, conducting ASC versus HC tests to identify disease‐specific structural differences, and HC versus HC tests to detect variability attributable to non‐disease factors. By combining these approaches, we aim to isolate robust, disease‐specific findings that transcend site‐specific and methodological variations, addressing the inherent challenges of heterogeneity in multicenter datasets and highlighting the complexity of studying a disorder as diverse as autism (Mottron and Bzdok 2020).

## 2 Materials and Methods

### 2.1 ABIDE I Dataset

The ABIDE I database comprises magnetic resonance images shared by 20 international centers. For our analysis, we accessed a dataset consisting of 1032 images, with 527 images corresponding to HC and 505 images representing individuals with ASC, as summarized in Table 1. The brain images of each subject sourced from the ABIDE I database underwent preprocessing and segmentation procedures, with only the complete GM map of each image being utilized. Subsequently, the brain volumes were partitioned into 116 ROI using a brain atlas established by the automated anatomical labeling (AAL) method (Tzourio‐Mazoyer et al. 2002). Within the article (Sun et al. 2009), there is a table (Table 2) which provides the names and corresponding numbers, according to the predefined AAL order, of the 116 ROI that are employed in the study.

**TABLE 1:** Number of brain images contributed by each center, categorized according to their condition, specifically control (HC) or autism (ASC).

Sites	n		Sex				Age			
	HC	ASC	HC		ASC		HC		ASC	
	M	F	M	F	Mean	SD	Mean	SD		
CALTECH	18	19	14	4	15	4	28	10.9	27.4	10.3
CMU	13	14	10	3	11	3	26.8	5.7	26.4	5.8
KKI	28	20	20	8	16	4	10	1.2	10	1.5
LEUVEN_1	15	14	15	0	14	0	23.3	2.9	21.9	4.1
LEUVEN_2	19	15	14	5	12	3	14.2	1.5	13.9	1.3
MAX_MUN	28	24	27	1	21	3	24.6	8.8	26.1	14.9
NYU	100	75	74	26	65	10	15.7	6.2	14.7	7.1
OHSU	15	13	15	0	13	0	10.1	1.1	11.7	2.3
OLIN	14	20	14	0	17	3	16.9	3.7	16.7	3.4
PITT	26	30	22	4	26	4	18.3	6.1	18.9	7.2
SBL	15	15	15	0	15	0	33.7	6.6	35	10.4
SDSU	22	14	16	6	13	1	14.2	1.9	14.7	1.8
STANFORD	17	18	13	4	14	4	10	1.7	9.9	1.6
TRINITY	25	22	25	0	22	0	17.1	3.8	17.5	3.7
UCLA_1	30	41	27	3	35	6	13.4	2.1	13.1	2.6
UCLA_2	13	13	11	2	13	0	12.3	1.1	12.7	1.9
UM_1	54	53	37	17	45	8	14	3.2	12.8	2.4
UM_2	21	13	20	1	12	1	16.7	4	14.9	1.6
USM	26	44	26	0	44	0	22.2	8.2	23.6	8.5
YALE	28	28	20	8	20	8	12.7	2.8	12.7	3
**Total**	**527**	**505**	**435**	**92**	**443**	**62**	**354.2**	**83.5**	**354.6**	**95.4**

**TABLE 2:** P‐values calculated for the regions of the NYU center. Only the regions that exhibited a frequency of significant differences within the confidence interval are included.

Regions	p‐value	Regions	p‐value
Precentral_L	0.3337	Occipital_Mid_R	0.3566
Precentral_R	0.4096	Occipital_Inf_R	0.4216
Supp_Motor_R	0.4296	Fusiform_R	0.4316
Calcarine_L	0.4945	Postcentral_L	0.3177
Calcarine_R	0.4705	Postcentral_R	0.3037
Cuneus_R	0.3317	Parietal_Inf_L	0.3656
Lingual_L	0.4575	Precuneus_L	0.2757
Lingual_R	0.4146	Precuneus_R	0.3776
Occipital_Sup_R	0.3347	Paracentral_Lob_L	0.3576
Occipital_Mid_L	0.3487	Temporal_Sup_L	0.4046

### 2.2 Preprocessing and Outliers Detection

The magnetic resonance images (MRI) obtained from the ABIDE I database were subjected to processing and segmentation using the SPM software. While SPM was primarily developed for functional imaging, it also offers functionalities for spatial realignment, smoothing, and normalization in the standard T1‐weighted image space. The entire process was conducted utilizing the computational anatomy toolbox (CAT12) within SPM (Gaser et al. 2024). The preprocessing steps, including co‐registration and segmentation, as well as the specific parameters employed at each stage, can be summarized as follows:

1. Preprocessing
  - The process used tissue probability maps to guide the analysis. The International Consortium for Brain Mapping (ICBM) provides these maps, which are derived from 452 T1‐weighted scans that were aligned in MNI space, corrected for scan inhomogeneities, and classified into three tissue types: GM, WM, and cerebrospinal fluid (CSF). The resulting maps were used to estimate a non‐linear deformation field that best aligns the tissue probability maps with each individual subject's image. The images were resized to 79 × 95 × 79 voxels with voxel sizes of 2 mm (sagittal) × 2 mm (coronal) × 2 mm (axial).
  - A mutual information affine registration with the tissue probability maps was used to achieve approximate alignment.
  - Spatial normalization was based on a high‐dimensional Dartel normalization and used standard Dartel template provided by CAT12
2. Segmentation
  - For each tissue class (GM, WM, and CSF), the intensity distribution was represented using a certain number of Gaussians, 2 in this case. The use of multiple components per tissue allows to reckon partial volume effects and deep GM differing from cortical GM.
  - A very light bias regularization was performed to correct smooth, spatially varying artifacts that modulate the intensity of the images.
  - A spatial adaptive non‐local means denoising filter is applied to the data in order to remove noise while preserving edges. The smoothing filter size is automatically estimated based on the local variance in the image.
  - Skull stripping was performed using CAT12 tool and its templates.

As a result of the preprocessing and segmentation procedures, probability maps were generated for each MRI image in the database. These maps assigned values within the range of 0 to 1 to each voxel, representing the probability of that voxel belonging to specific tissues, such as WM, GM, or CSF. However, for the purpose of our study, only the GM map derived from the images will be used.

### 2.3 Statistical Parametric Mapping

SPM, or statistical parametric mapping (Friston et al. 1994; Penny et al. 2011), is a neuroimaging tool that utilizes voxel‐wise statistical analysis to examine and compare structural and functional brain data in relation to different conditions or tasks. The statistical analysis of MRI data employs a univariate mass approach based on general linear model (GLM). This approach entails the specification of a GLM design matrix, which in our case involves utilizing a two‐sample t‐test to divide the MRI images into two equally‐sized groups. The images are smoothed by a Gaussian filter of size 8 FWHM. Subsequently, the model is estimated through the classical method, employing restricted maximum likelihood to estimate the parameters. This estimation assumes that the error correlation structure is uniform across all voxels. Following the estimation, specific parameter profiles are subjected to testing using T statistics with contrasts assigned values of +1 and −1 for each respective image class. To mitigate the risk of false positive results in the context of multiple comparisons, a whole‐brain cluster‐level family‐wise error (FWE) correction was applied, maintaining a significance level (α) of 0.05, rejecting the null hypothesis if p< 0.05. In this approach, an initial cluster‐forming threshold is applied at the voxel level (here, p < 0.001 uncorrected). Contiguous voxels exceeding this threshold are grouped into clusters, and the probability of observing clusters of that size under the null hypothesis is estimated using random field theory. This procedure controls the family‐wise error rate across the entire brain, accounting for spatial correlations among voxels, and provides cluster‐level p‐values that indicate whether each cluster is significant while correcting for multiple comparisons. Compared to Bonferroni correction, cluster‐level FWE is less conservative in spatially correlated neuroimaging data, balancing sensitivity to detect extended regions of effect with rigorous control of FPs (Friston et al. 1994).

**FIGURE 1:**
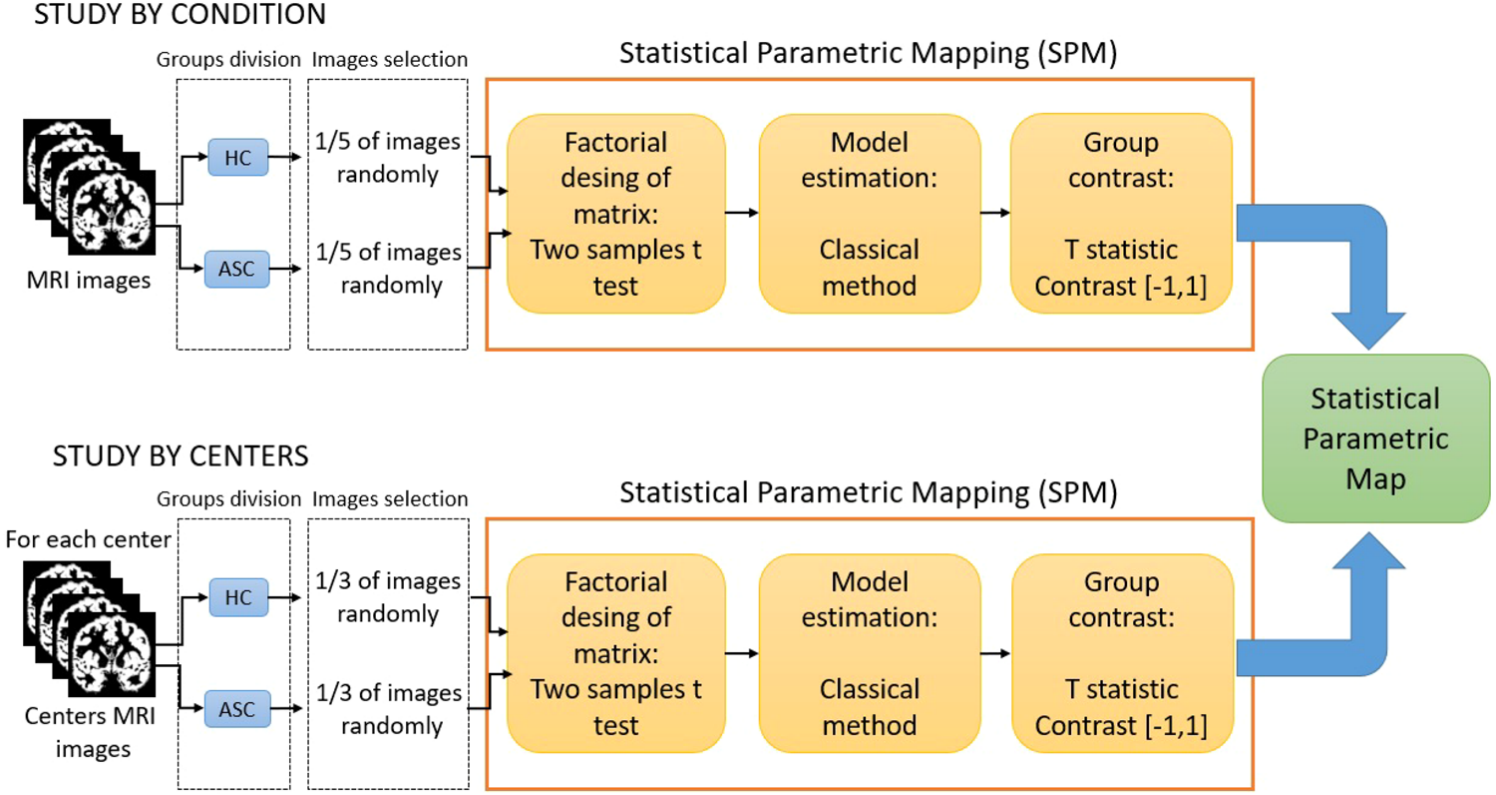
The block diagram illustrates the MRI analysis process using Statistical Parametric Mapping (SPM) and its three main steps. The diagram specifically focuses on the comparison between HC and individuals with Autism Spectrum Condition (ASC). For the comparison between healthy controls themselves (HC vs. HC), the group division involves creating two separate HC groups while maintaining the remaining stages of the analysis process.

A voxel‐wise threshold was set to 0.05 and the extent of the minimal cluster size was set to 0, as exploratory values. Figure 1 presents a block diagram illustrating the analysis process with SPM, highlighting the main steps involved.

### 2.4 Statistical Agnostic Mapping

SAM (Gorriz et al. 2021) is a nonparametric ML method used to evaluate neuroimaging data at the voxel or regional level. It addresses the issue of unstable risk estimates that arise from limited sample sizes by employing cross‐validation (CV) techniques in ML (Varoquaux 2018; Gorriz et al. 2024). Moreover, SAM offers an alternative approach for mapping p‐values that are corrected for FWE under *the worst case*, ensuring the reliability of inferential statistics for hypothesis testing. The methodology of SAM is grounded in the data and employs concentration inequalities to test opposing hypotheses or compare different models. SAM generates activation maps similar to those obtained through voxel‐wise analysis in SPM, but with a focus on regions of interest. It has been extensively developed and tested in scenarios characterized by a small sample‐to‐dimension ratio and varying effect sizes, including large, small, and trivial effects(Jimenez‐Mesa et al. 2023, 2024; Jimenez‐Mesa, Peñalver, et al. 2022; Jimenez‐Mesa, Castillo‐Barnes, et al. 2022; Segovia et al. 2023).

#### 2.4.1 Feature Extraction and Selection

The SAM procedure can be summarized into several steps as follows:

1. The data will be prepared by designing a comparison between groups, referred to as the design matrix. ROI will be selected for analysis, focusing on specific areas within the subjects' brains.
2. For each ROI, a feature extraction and selection (FES) stage will be conducted to derive the feature space. This involves identifying relevant features that contribute to the classification task. A support vector machines (SVM) will be trained using empirical risk minimization (ERM) to estimate the replacement error.
3. The empirical error or precision will be calculated based on the trained SVM model. Additionally, the true precision will be determined in the worst‐case scenario with a probability of 1 − α. This step helps assess the accuracy and reliability of the results.
4. The z‐test statistic will be computed for each true precision to evaluate their significance. This statistical test assesses whether the observed results deviate significantly from what would be expected by chance, providing insights into the meaningfulness of the findings.

**FIGURE 2:**
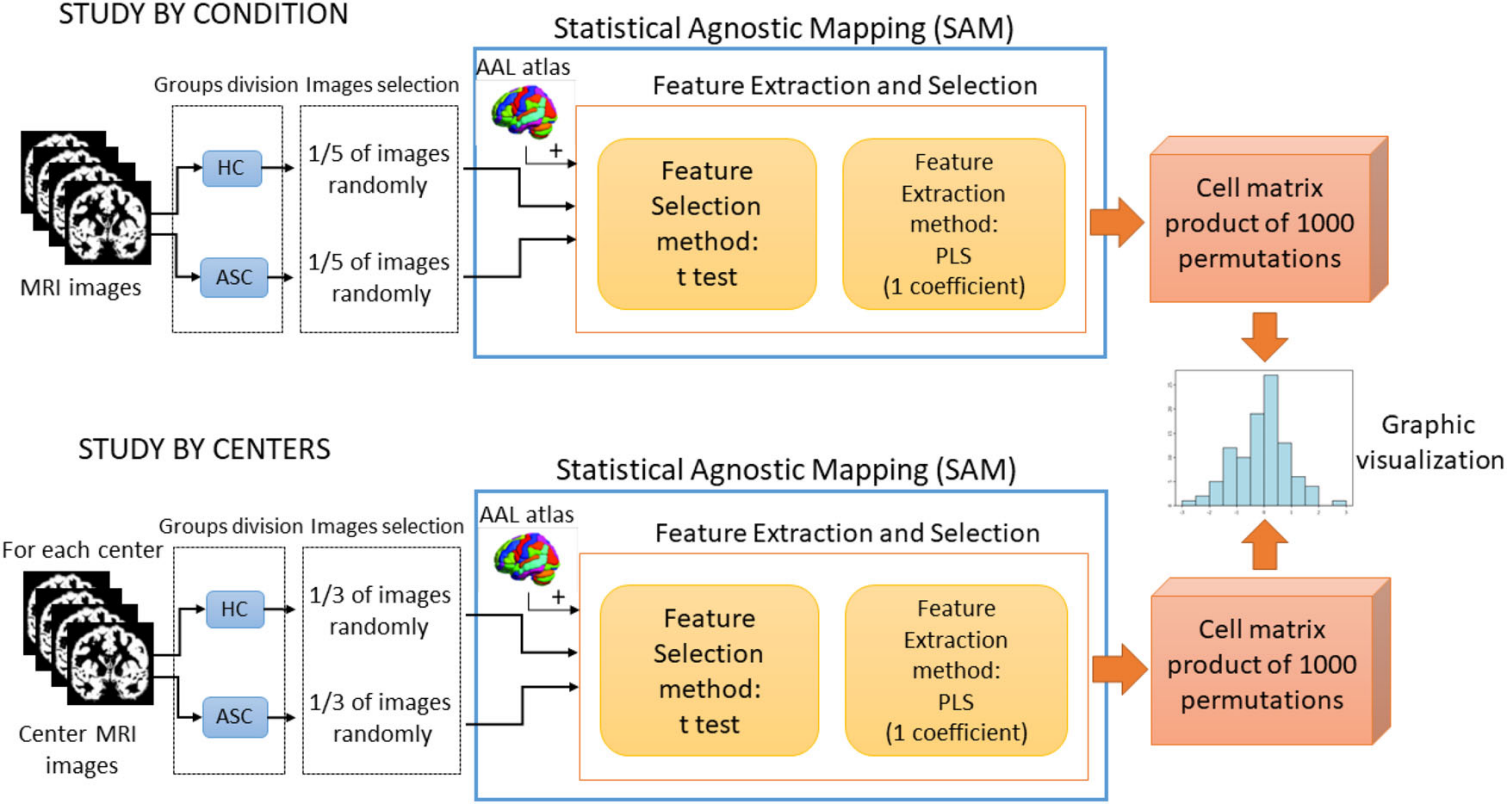
The block diagram illustrates the MRI analysis process incorporating Statistical Agnostic Mapping (SAM) and the feature extraction and selection stage. Specifically, the diagram represents the comparison between HC and individuals with ASC. As for the comparison between HC themselves (HC vs. HC), the group division will involve creating two separate HC groups, while retaining the remaining stages of the analysis process.

These steps collectively form the SAM procedure, allowing for the identification and evaluation of significant differences between groups in the selected regions of interest. In this study, default values are used for the SAM implementation (v1.0; SiPBA (2024)). The input data for SAM will consist of images divided into two classes, namely HC class images versus ASC class images; and HC class images versus HC class images. To select relevant features, we will employ a t‐test. Within each ROI, SAM will calculate the t‐test statistic value for each voxel, and we will select the 50 voxels (features) with the highest t‐test values. This threshold of 50 voxels has been determined based on the consideration of images with a voxel size of 2 × 2 × 2 ${\text{mm}}^{3}$ and an atlas comprising 116 regions, as it has consistently yielded favorable outcomes in previous studies(Gorriz et al. 2021). Partial least squares regression (PLS) will be used for feature extraction(Krishnan et al. 2011), with a single PLS dimension being employed (see Appendix A.2). PLS has demonstrated its effectiveness in capturing relationships between brain activity and experimental design or behavioral measures within a multivariate framework. The complete analysis pipeline, which incorporates FES, is illustrated in Figure 2 as a block diagram.

### 2.5 Statistical Analysis by Permutation Testing

In this article, we employ a two‐sample statistical analysis using permutation tests (Pesarin and Salmaso 2010; Good 2000) to identify brain regions that exhibit statistically significant differences. The permutation test is used to evaluate whether the observed classification accuracy T is significantly better than what could be expected by chance. To calculate the p‐value, we compare the observed accuracy with the accuracies obtained under many random label permutations ($T_{\pi }$). The numerator of the equation counts how many times the permuted accuracies are greater than or equal to the observed accuracy, and one is added to include the observed result itself. The denominator is the total number of permutations plus one, ensuring validity of the test. This way, the p‐value represents the probability of achieving an accuracy at least as high as the observed one purely by chance.

(1) $$p\_\text{value}=\frac{[\#T_{\pi }\geq T]+1}{M+1}$$

where M is the number of permutations.

The study involves analyzing brain images using two different approaches: by center and by condition. In each case, two types of comparisons will be conducted: between individuals with autism and controls (HC vs. ASC), and between controls themselves (HC vs. HC). When analyzing by center, these comparisons will be performed individually for each center. The comparisons of primary importance are those between HC and ASC, as they involve distinct classes of individuals and determine the presence of significant differences between the groups. However, the comparison of HC versus HC is included to identify any regions that may exhibit significant differences unrelated to the disorder, possibly due to chance or other external factors.

To conduct the aforementioned comparisons, a permutation test will be employed, with M=1000 permutations performed for each case(Ojala and Garriga 2009). The permutation test is a statistical significance test used to examine differences between groups. It involves calculating the statistic value for all possible rearrangements of observations within the different groups. In each permutation, the brain images will be divided into two groups of equal sizes: HC and individuals with ASC or two HC groups.

### 2.6 Sample Size and Power Calculations

**FIGURE 3:**
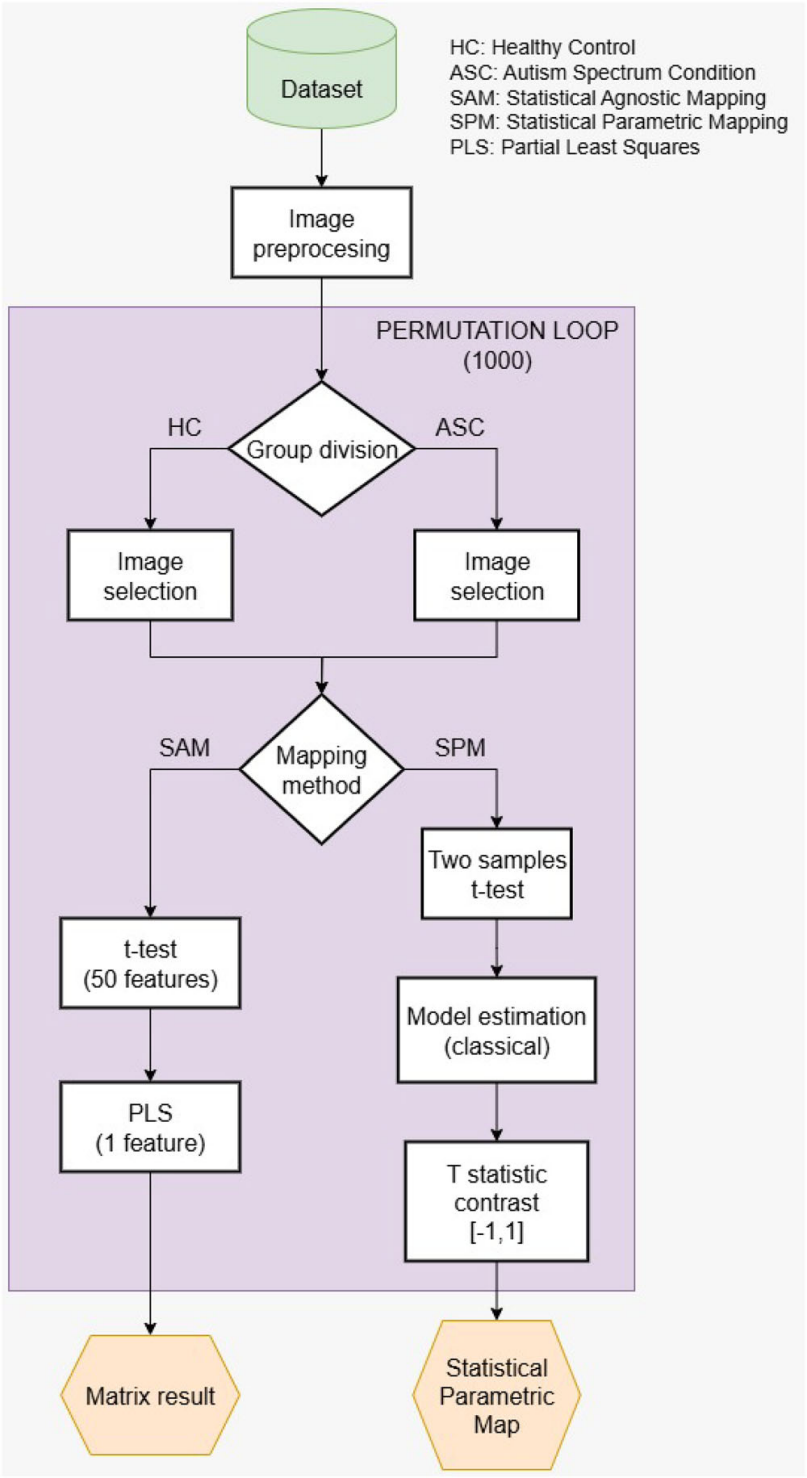
Block diagram about ABIDE I dataset analysis by permutation test with both SAM and SPM mapping methods.

A specific number of images will be randomly selected from each group for the analysis, assuming the sample size needed to detect the minimum detectable effect. In the case of the study based on the condition of the patients (HC vs. ASC), approximately 1/5 of the total number of images contributed by each group will be taken, resulting in 100 images from each group. Conversely, for the study conducted by centers, 1/3 of the images from each group will be selected individually at each center. This ensures a balance between the sample size and the available dataset, creating distinct subgroups in each permutation. The varying sample sizes are a result of the total number of samples available in each case, with the study by centers having a smaller number of available images. In each permutation, the selected images will be compared to one another using the respective mapping method being employed. This involves region‐to‐region comparisons using SAM and voxel‐level comparisons using SPM. Figure 3 illustrates the framework of the analysis process.

## 3 Experimental Results

The initial data analysis will be performed using the data‐driven SAM method. The objective is to take advantage of the excellent control over FPs provided by this method, in contrast to the optimistic cluster wise inference of parametric methods (Eklund et al. 2016).

### 3.1 SAM Analysis

First, the analysis will be conducted based on the condition, where all images contributed by each center will be grouped together, distinguishing between ASC and HC. After applying FWE correction to the spatial discriminance map obtained through machine learning (SAM output) using the upper bound correction, no FPs are expected due to the multiple comparison problem, as SAM evaluates the worst‐case scenario and only 116 values are considered. Using 1000 permutations and a confidence level of 0.95, we expect approximately 50 FPs by chance under the null hypothesis. Therefore, regions exhibiting more than 50 significant differences will be considered true positives (TPs), as they fall within the predetermined 95% confidence interval for the HC versus ASC comparison. The results indicate the presence of numerous regions showing a notable number of significant differences.

Surprisingly, even in the HC versus HC comparison, where any significant difference should be regarded as a false positive, regions displaying substantial differences are observed. Notably, both comparisons reveal the same significant regions with highly similar values, suggesting that many of these differences are not solely attributable to the patient's condition but rather to other factors. To investigate whether these differences may be attributed to the different imaging centers that contributed the data, a subsequent analysis will focus on the individual centers.

**FIGURE 4:**
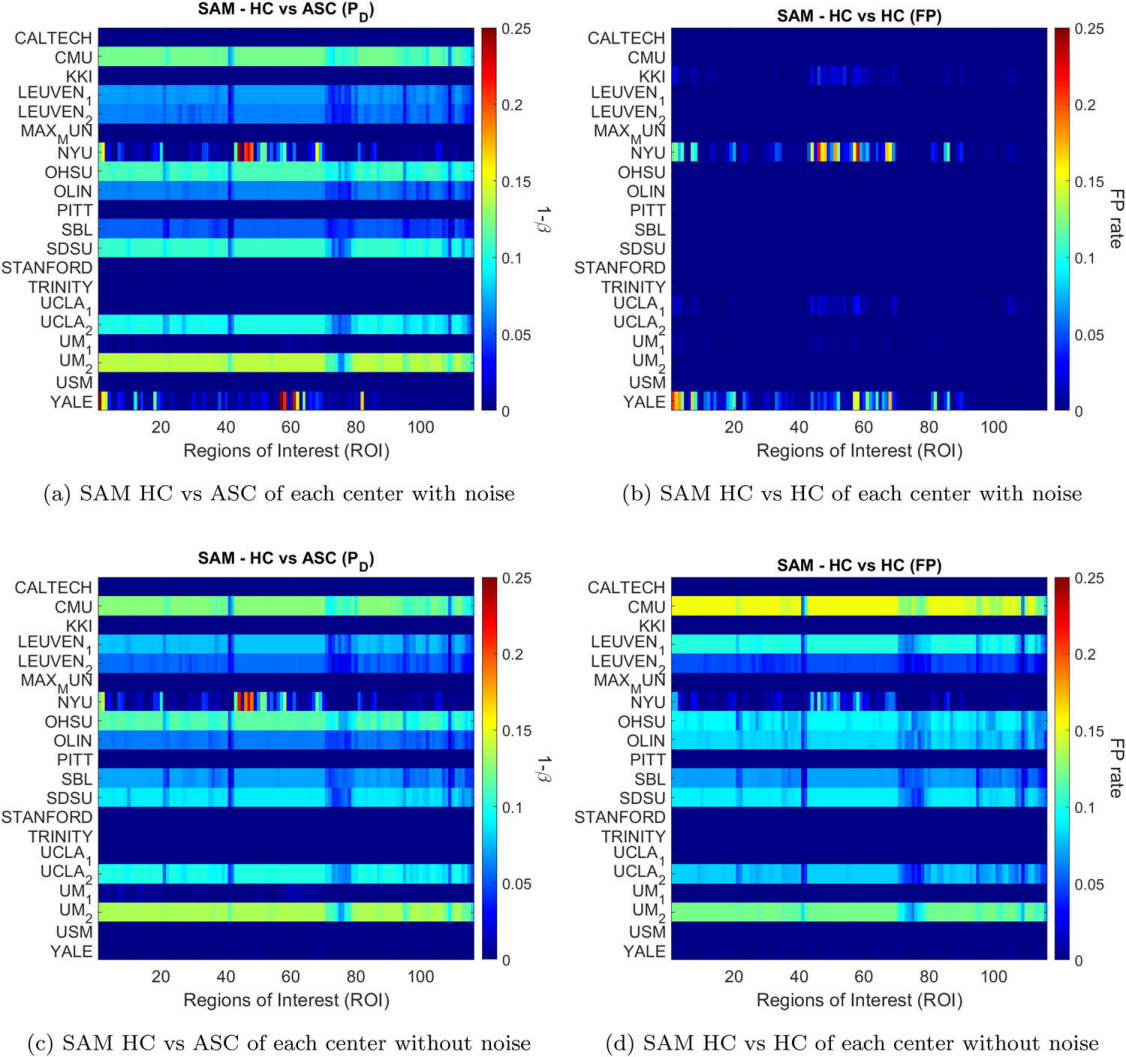
Estimated probability of detection ($P_{D}$) and false positive (FP) through permutation testing using the SAM mapping method. Each colormap rectangle represents one of the 116 brain regions defined in Table 2 of Sun et al. (2009). The color map depicts the probability of detection of significant differences observed in the regions during the permutation test.

The permutation test, combined with SAM, was performed separately for each center in both comparisons (see Figure 4a,b where we display “probability of detection”). The results reveal high variability across centers, with three distinct patterns of behavior: (i) Centers where no significant differences are observed in any region, regardless of the comparison, suggesting an absence of distinguishing brain regions between ASC and HC. Interestingly, even random factors fail to produce minimal differences in these centers. (ii) Centers where the HC versus HC comparison shows no significant differences, yet the HC versus ASC comparison exhibits significant differences across nearly all regions with similar frequencies. This unusual pattern, where almost every region shows significant differences, is highly improbable and likely attributed to small sample sizes (<17 scans) in relation to the ‘magical’ number proposed in Friston (2012). (iii) Centers such as NYU, UM 1, and YALE present a more expected outcome, with a mix of regions showing significant and non‐significant differences in both comparisons.

**FIGURE 5:**
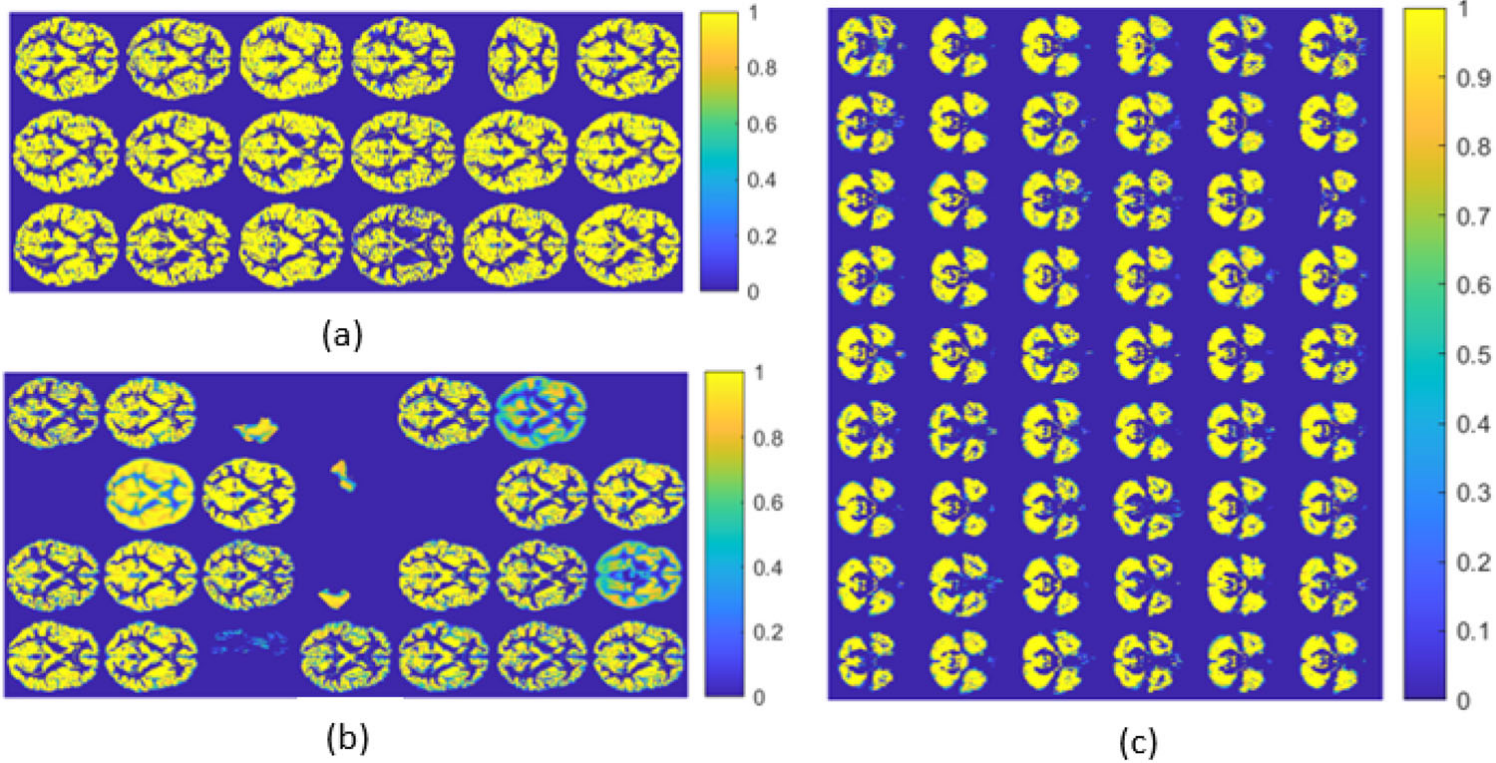
Brain mosaics from different centers to assess the presence of failed pre‐processed images. Specifically, the mosaics of three centers with distinct issues in their images are shown: (a) CALTECH's cerebral mosaic, (b) YALE's cerebral mosaic, and (c) UM_1's cerebral mosaic.

The initial analysis was performed without accounting for the presence of failed pre‐processing images, as shown in Figure 5. Their presence can be attributed to errors in the acquisition or pre‐processing pipeline, and affects every group randomly. Therefore, the balance in age or center is not affected by excluding them. The impact of incorporating defective images in the region detection process can be assessed by subsequently removing them from the dataset, thereby assessing the overall influence of noisy data.

#### 3.1.1 Removal‐of‐Outliers Analysis

The identification of outlier images followed the steps: (1) visual and automated QC: images flagged by visual inspection (motion artifacts, severe segmentation failures) were excluded; (2) statistical outlier detection: for each voxel we computed *z*‐scores and flagged voxels exceeding |z| > 5 were computed. The percentage of flagged voxels in the dataset ranged from 0% to 12% voxels per image. A conservative percentage threshold was determined as exclusion criteria: if a subject contained more than 3% of flagged voxels the image was marked as suspicious, and confirmed afterward by visual inspection. Subjects flagged by both criteria were excluded, with a total of 21 images identified as unusable due to failed preprocessing.

Analyses conducted after excluding these images yielded results that were highly consistent with the original findings.

**FIGURE 6:**
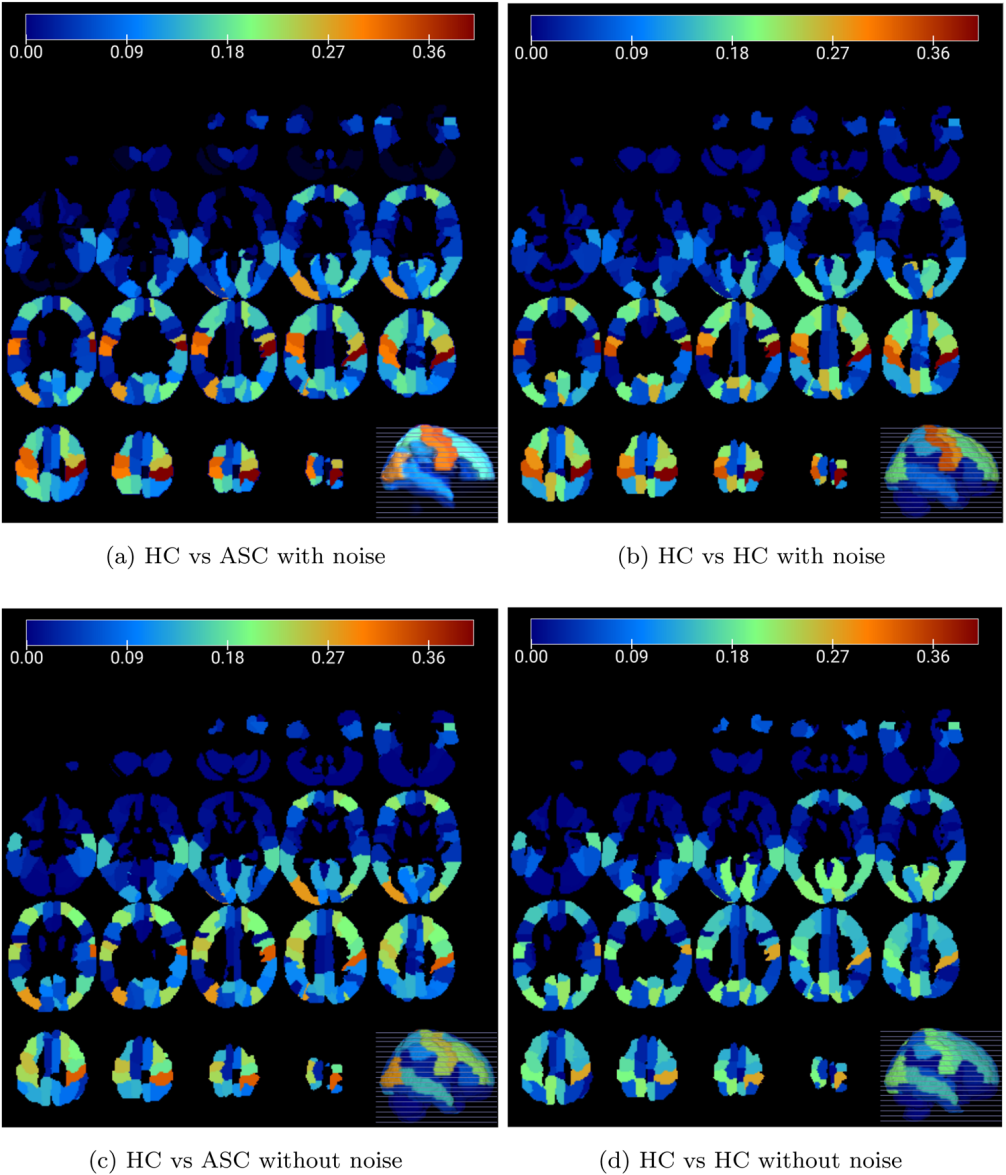
All center study using the SAM mapping method, highlighting the $P_{D}$ observed in each region during the comparisons HC versus ASC and HC versus HC from left to right, respectively. Above are the comparisons prior to the removal of flawed scans. Below, the same comparisons are presented after removing flawed scans.

**FIGURE 7:**
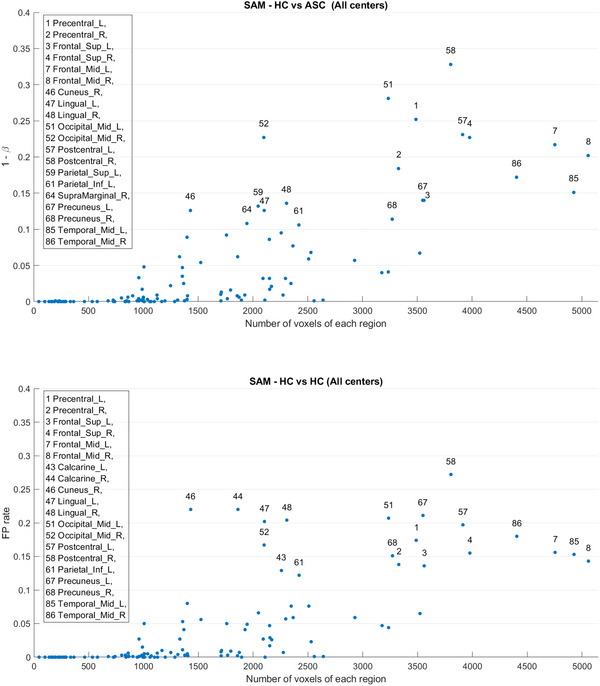
Upper image shows probability of detection $P_{D}$ obtained with the SAM mapping method in the all‐centers comparison. The vertical axis is expressed as 1−β, where β denotes the Type II error rate; thus, 1−β corresponds to statistical power. Lower image shows FP rate in the same conditions. Each point represents a specific brain region, with the most prominent ones highlighted.

Figure 6 presents the results of the analysis by condition. The regions showing significant differences remained largely consistent in both the HC versus ASC and HC versus HC comparisons. Notably, the frequency of these differences changed only minimally after the removal of noisy scans. To further investigate, Figure 7 replicates the results shown in panels (c) and (d) of Figure 6, but additionally includes voxel size information for each region. These results suggest a correlation between false positive rate and region size, even in the HC versus HC comparisons, indicating that the effect is not related to the ASC condition. This effect is stronger than the impact of including noisy data.

Analyzing by centers, removing failed pre‐processed images produced slight changes. The NYU center displayed variations in significance levels across different regions, whereas the UM_1 and YALE centers, showed no significant differences in the HC versus HC comparison and only minor, non‐significant differences in the HC versus ASC comparison (see Figure 4c,d).

The analysis by centers concludes that those with limited patient numbers lack sufficient sample sizes for meaningful insights, providing overly optimistic or pessimistic significance maps. In contrast, the NYU center, with its substantial patient count, offers robust potential for further study and more reliable outcomes.

#### 3.1.2 Study of NYU Center

**FIGURE 8:**
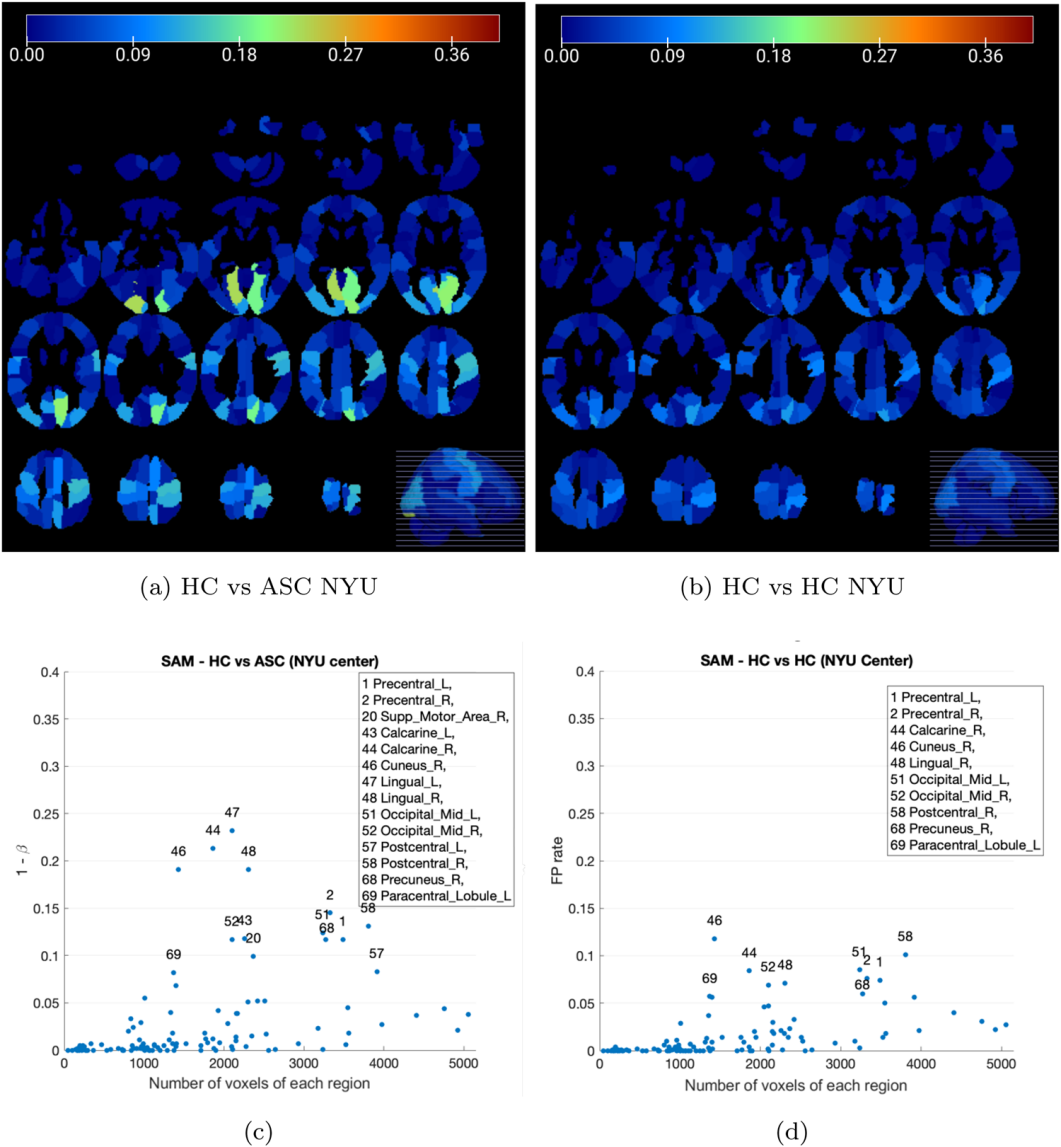
Brain regions with significant differences in both comparisons (a) HC versus ASC and (b) HC versus HC using the SAM permutation test after defective images removal in NYU. (c) Region voxel size versus estimated $P_{D}$ in the HC versus ASC comparison using SAM. (d) Region voxel size versus FP rate in the HC versus HC comparison using SAM. Each data point on the diagram represents a specific brain region, highlighting the most prominent ones.

The NYU center, with its large number of images, provides a robust dataset for focused studies. However, the results obtained from the permutation test in the NYU center reveal that the same brain regions consistently exhibit significant differences in both comparisons, while other regions consistently show no significant differences (Figure 8). Notably, posterior brain regions such as Calcarine_R, Cuneus_R, Lingual_L, and Lingual_R stand out prominently.

Considering the smooth changes in frequencies observed across different centers in Figure 4, it is plausible that a relationship exists between the size or number of voxels comprising each region and its significance. On the left in Figure 8c, we show a scatter plot comparing the voxel size of each region with the number of times it exhibits significant differences (statistical power if we assume an effect in the sample). A threshold of 0.05 has been set on the *y*‐axis (significance level). The scatter plot suggests a relationship between these two variables, indicating that the power of the test increases as larger regions are considered. It can be inferred that regions below 1000 voxels are never significant. These findings could highlight the importance of considering multivariate approaches and the size of brain regions when analyzing significance, as larger regions provide more reliable and informative results in neuroimaging studies.

Unfortunately, a similar effect on the rate of FPs is observed in the right part of the same figure (Figure 8d). This observation indicates that there is indeed a trivial effect present in the entire dataset, with effects correlated in both groups. Upon closer examination of the results from the permutations test, it becomes apparent that the brain region with the most significant differences in both comparisons occurs approximately 240 times out of the 1000 permutations. The calculated p‐values (average) for the brain regions in Table 2 were found to be higher than the predefined significance level of α=0.05 (see Table 2). Therefore, besides the abnormal rate of FPs, it can be concluded that similar brain regions in the NYU center images exhibited non‐significant differences in both comparisons. However, the surprisingly high rate of FPs in the HC versus HC comparison affects the computation of p‐values in the ASC versus HC study, as shown at the bottom of the table. Assuming the p‐values follow a normal distribution according to the empirical rule (68‐95‐99.7), the lack of evidence for rejecting the alternative hypothesis does not prove that the effect does not exist at the significance level (95%).

### 3.2 Analysis With SPM

The initial study was conducted by grouping all the images based on patient condition, disregarding the centers to which they belong. The images were divided into two groups: ASC and HC. The database analysis using SPM is conducted through a two‐tailed test, aiming to detect differences in means between the groups. For the assignment of contrasts (+1/−1), both tails of the test were examined, assigning in one study HC(−1) < ASC(+1), and in the other ASC(−1) < HC(+1). The results of the significant voxel count obtained for each study are summarized in Table 3. The analysis was conducted on the complete database and at the center with the largest sample size (NYU).

**TABLE 3:** Number of significant voxels obtained after analyzing the database with SPM by examining both tails of the test.

	Database HC < ASC	Database ASC < HC	NYU HC < ASC	NYU ASC < HC
Voxels	10	197	13	100

As observed in the table, the analysis for ASC < HC reveals significantly more voxels than for the opposite contrast, suggesting a decrease in gray matter in patients with ASC, as indicated by some studies in the literature (Li et al. 2019; Huemer et al. 2016; McAlonan et al. 2005, 2008). Therefore, the comparison of the analyses with the results obtained through SAM and the subsequent representation will be based on the ASC < HC contrast.

**FIGURE 9:**
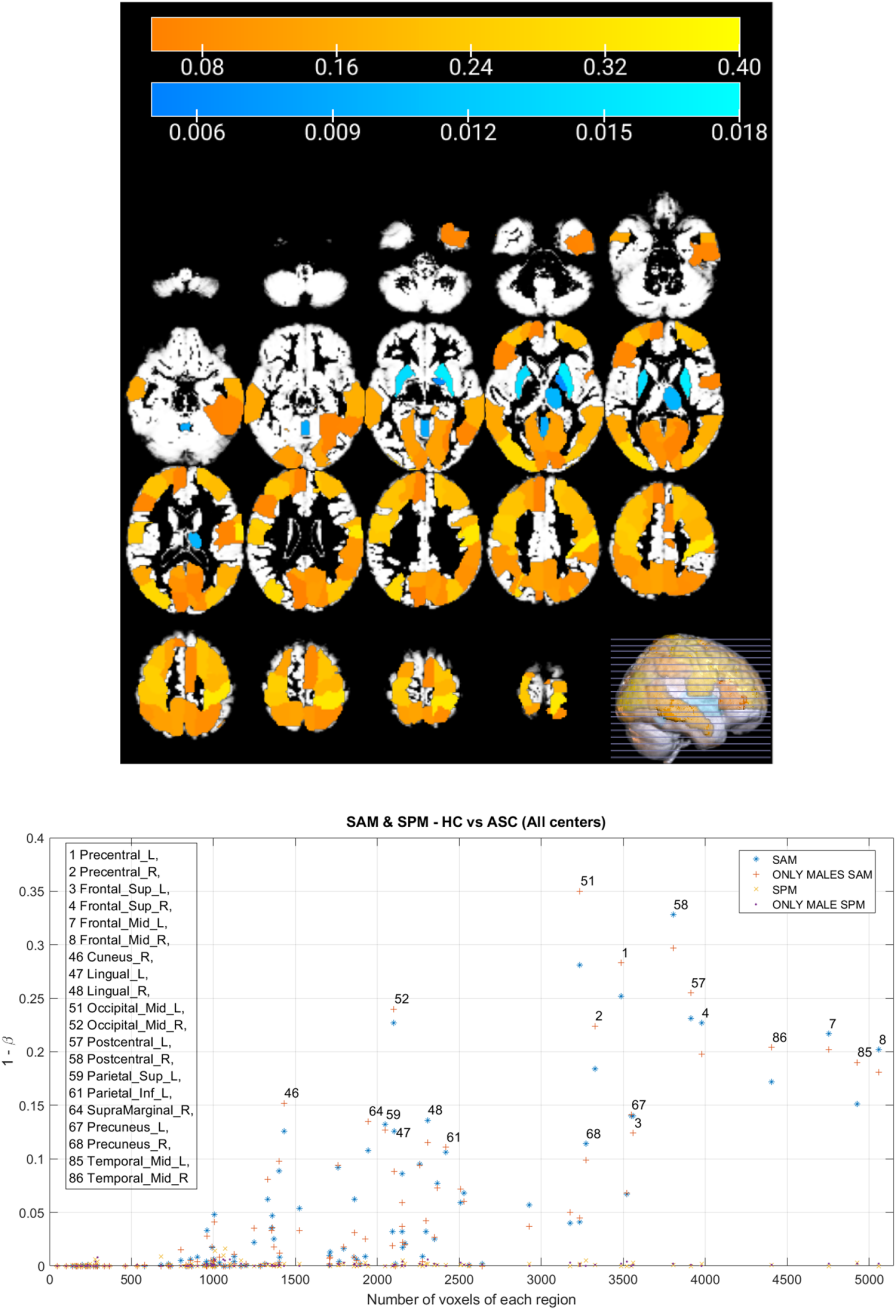
SAM and SPM results for HC versus ASC all centers comparison. In the upper image, it is represented in orange the $P_{D}$ at each brain region detected by SAM statistical method and in blue the $P_{D}$ detected by SPM. In the lower image, the SAM and SPM comparison is presented, including their respective analyses with male subjects only.

At the bottom of Figure 9, we computed $P_{D}$ of the SPM voxelwise statistical framework and compared it to that obtained with the SAM method. As clearly shown in the figure, SPM did not provide the nominal value of 0.05, indicating an overly super‐conservative method. This behavior is indeed persistent in studies with larger sample sizes, as shown in the next section. Following these results, the p‐value analyses did not reveal any evidence to reject the null hypothesis at the significance level.

#### 3.2.1 Study by Center

The SPM analysis failed in Group I centers (CALTECH, KKI, LEUVEN 1, LEUVEN 2, MAX MUN, OLIN, SBL, SDSU, STANFORD, TRINITY, USM) due to the limited number of samples available for analysis. However, the NYU center, which has the largest number of images, demonstrates minimal voxels with differences and a significantly lower frequency of regions showing significant differences. In fact, the regions exhibiting differences in the center of NYUNYU fall well below $t_{50}$ threshold, which was previously established as the nominal value or the expected value of the FPs under the null hypothesis. This observation holds for both the study by condition and the study by centers using SPM.

**FIGURE 10:**
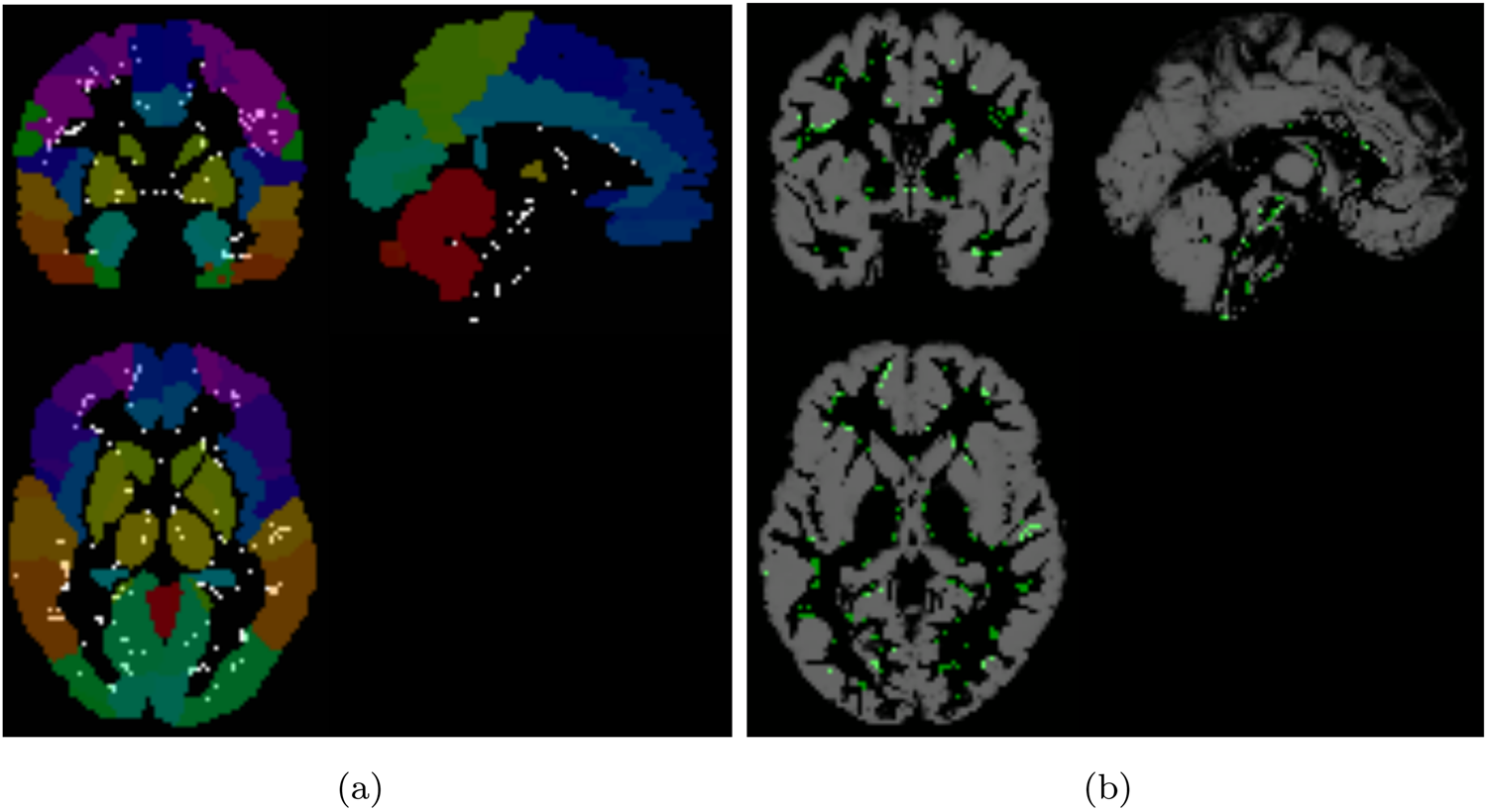
SPM t‐map in ASC versus HC comparison of OLIN center, compared to the AAL atlas (a), and compared to the grey matter (GM) map (b).

In contrast, some limited samples exhibit a higher number of voxels showing differences after contrast analysis. Taking the OLIN center as an example, we can observe the voxels that have been identified as different after the permutation test using SPM in Figure 10, superimposed on the image provided by the AAL atlas. These voxels, located near the edges of different regions, can be attributed to registration artifacts, which can arise from anatomical variations among subjects or discrepancies between the atlas‐defined regions and the detected regions during image registration. In addition, some differences are concentrated in the brain stem, as shown in the same figure.

**FIGURE 11:**
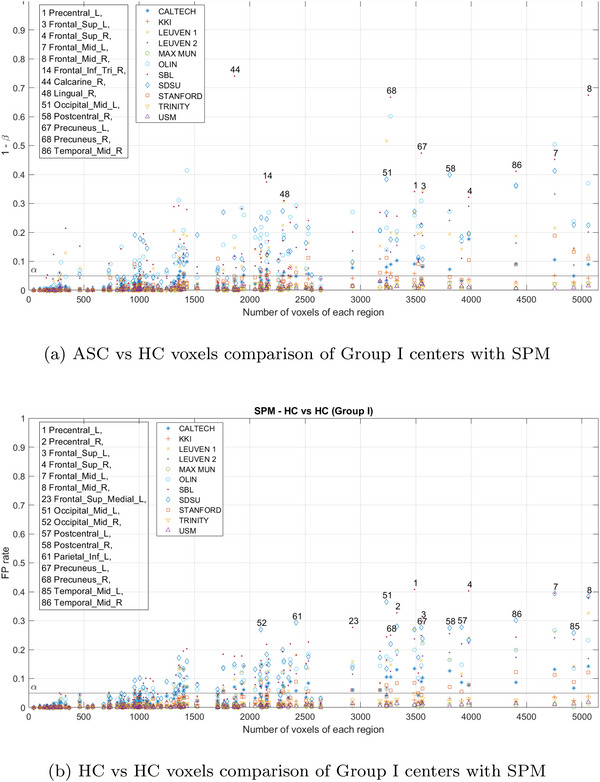
Estimated $P_{D}$ (upper) and FP rate (lower) with SPM during the Group I centers analysis. Each point on the diagram represents a specific brain region, highlighting the most prominent ones.

Figure 11 shows the frequency of significant voxels in various brain regions following the SPM contrast for centers with limited sample sizes (Group I). In particular, these centers exhibit relatively high voxel frequencies and disparities between regions. Approximately, 25% of the permutations failed to produce results in this group due to the limited number of available samples. Consequently, it can be concluded that the large number of voxels detected in these centers is not attributable to the identification of a specific region marking differences between individuals with autism and healthy controls. Instead, it likely stems from center‐specific characteristics, such as sample realization, confounding effects, etc.

**FIGURE 12:**
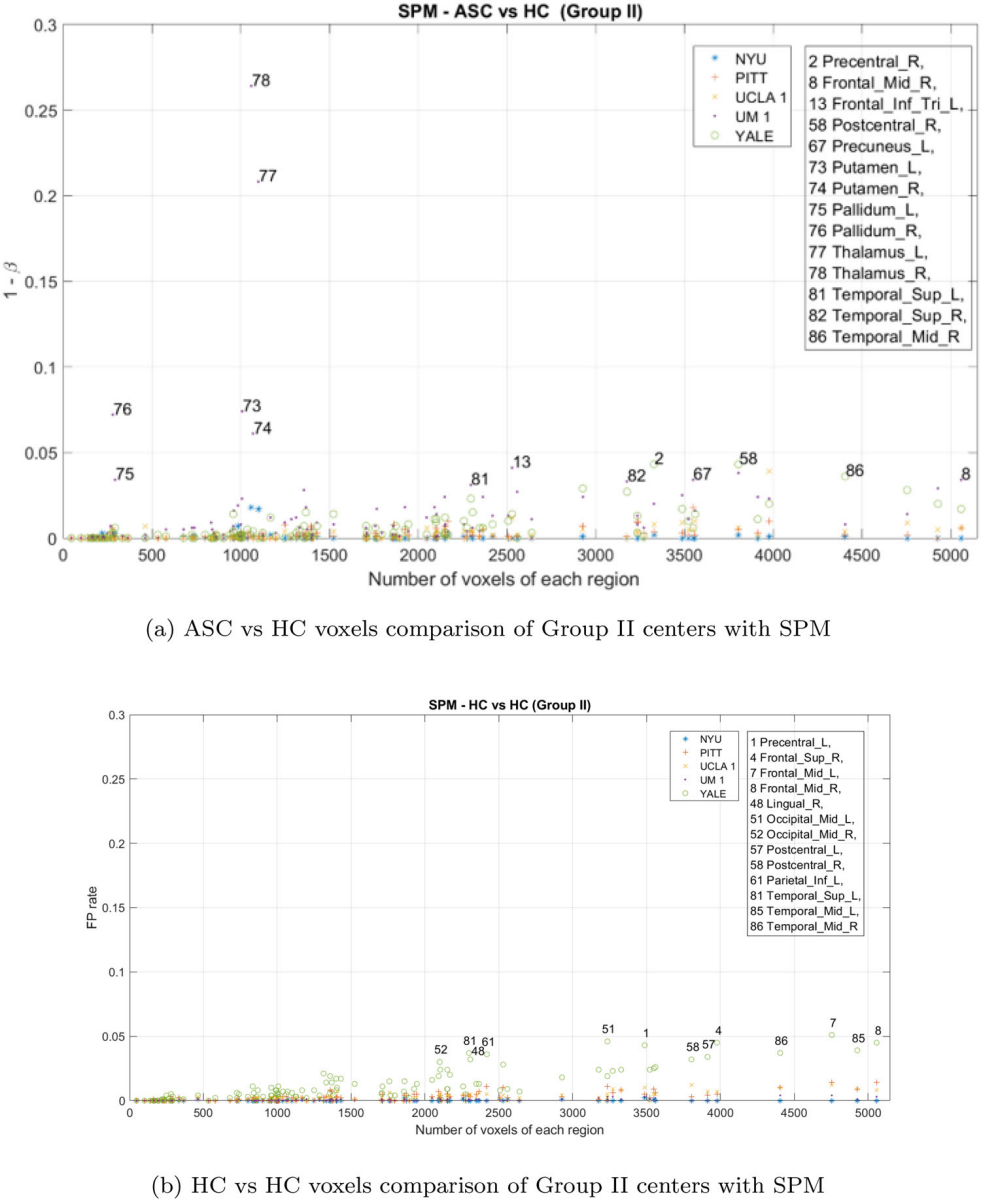
Estimated $P_{D}$ (upper) and FP rate (lower) with SPM during the Group II centers analysis. Each point on the diagram represents a specific brain region, highlighting the most prominent ones.

Figure 12 shows the results on centers with larger sample sizes (Group II: NYU, PITT, UCLA_1, UM_1, and YALE). Although there are regions that exhibit a substantial number of voxels, these completely vanish in the analysis across all centers.

After analyzing the images with SPM, both by condition and by center, it can be concluded that SPM does not identify any region or voxel that exhibits statistically significant differences at α=0.05. Therefore, based on the results obtained, it could be inferred that there are no specific brain regions or voxels that can be considered reliable indicators for distinguishing between individuals with autism and neurotypical individuals. Taking into account all the issues and artifacts found in the analyzed datasets, this is rather a consequence of the super conservative behavior of voxelwise statistical inferences in the SPM method.

## 4 Discussion

In this comprehensive investigation, we examine the outcomes derived from image analysis conducted on the ABIDE I database utilizing SAM and SPM mapping methodologies. Our analysis will evaluate the degree of concordance or divergence among these methodologies. Particular emphasis is placed on two primary dimensions: (1) by condition, that is, the real differences present in the ASC group, by comparing them to the artificial HC versus HC comparison (2) by acquisition site, that is, the influence of the site (NYC, PITT, UCLA,…) with its corresponding acquisition and processing differences, in the findings. Following this, we evaluate the alignment of our findings with those documented in prior research, if any.

### 4.1 Study by Condition

SAM analysis revealed the presence of brain regions with a notable number of significant differences for both comparisons (HC vs. ASC and HC vs. HC). However, the observed differences did not reach a sufficiently high level compared to the number of permutations conducted, thereby precluding the identification of any decisive regions as shown in the p‐value analysis. Indeed, upon fixing the significance level and calculating the p‐value for each region, it was determined that none of them exhibited statistically significant differences, although in this case we prefer to claim that there is no sufficient evidence in support of the alternative hypothesis. Similarly, the analysis performed with SPM demonstrated a scarcity of significant voxels in the T‐maps. The frequency of regions exhibiting differences at voxel level during the 1000 permutations were even below the nominal level (50), affirming the absence of statistically significant differences in any particular region using this over‐conservative method.

Figure 9 provides a comparison of SAM and SPM results, visualizing regions identified as different by SAM along with their corresponding frequency values using an orange color map, and highlighting the number of voxels that manifested differences after the SPM contrast using a blue color map. SAM highlights regions as Postcentral_R, Postcentral_L, Occipital_Mid_L, Occipital_Mid_R, Precentral_L, among others. Meanwhile, SPM highlights regions as Putamen_L, Putamen_R, Thalamus_R, Vermis_4_5, among others. Notably, the highest concentration of voxels detected by SPM was observed in regions where SAM indicated no or minimal differences, while regions exhibiting more significant differences according to SAM showed scarce significant voxels. Consequently, both methods did not individually establish statistically significant differences but contradicted each other in identifying regions with great disparities.

### 4.2 Study by Acquisition Sites

Both SAM and SPM methods revealed the need to differentiate the NYU center from the remaining centers in the study. In both methods, some centers have regions with significantly higher frequency or voxel count compared to NYU or the study conducted based on the subjects' condition. However, it is highly likely that these results, particularly in SAM where the values among centers were similar, stem from the limited number of images available for each center. The limited number of images, regardless of the mapping method employed, contributes to errors that compromise the reliability of the obtained results. Conversely, when the images from all centers are aggregated and analyzed as a whole (study by condition), the differences found between groups are diluted.

Regarding the NYU center, both SAM and SPM mapping methods exhibit remarkably similar results compared to the results obtained from the study conducted based on the subjects' condition. This can be attributed to the fact that NYU contributes the largest number of samples, making it the most suitable center for in‐depth examination among the 20 centers comprising the ABIDE I database.

In SAM, the NYU center demonstrates numerous brain regions that exhibit differences exceeding 100 occurrences during the 1000 permutations. In particular, during the HC versus ASC comparison, which is of particular interest, the regions Calcarine_R, Cuneus_R, Lingual_L, and Lingual_R emerge as the regions with the most significant differences, with two of them exceeding 200 out of 1000 occurrences. However, SPM analysis reveals a minimal number of voxels with differences after the contrast in the NYU center. The regions Hippocampus_R, Thalamus_L, and Thalamus_R exhibit the highest concentration of these differing voxels, while SAM either indicates no differences or minimal distinctions in these areas. Consequently, it can be concluded that both mapping methods contradict each other by identifying entirely distinct regions as positive. Nevertheless, both approaches reach the same statistical conclusion from the analysis of p‐values.

**FIGURE 13:**
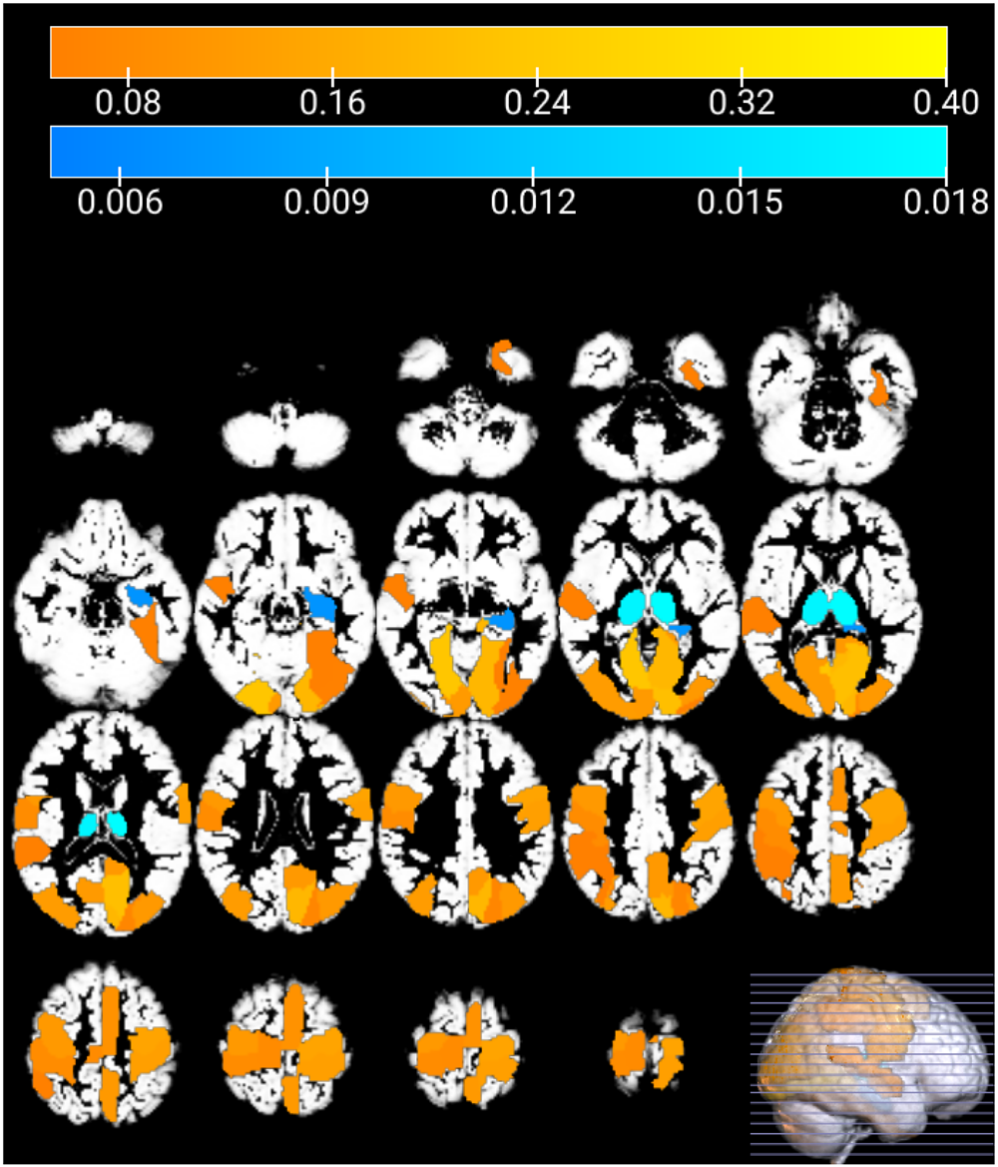
Comparison of SAM and SPM results for HC versus ASC comparison at NYU center. In orange, the differences in brain regions detected by SAM statistical method. In blue, the differences detected by SPM.

Figure 13 visually presents the comparison between SAM and SPM results, illustrating the regions identified by SAM as having significant differences in each permutation along with their corresponding frequency denoted by an orange color map, as well as the most significant voxels detected by SPM in each permutation, denoted by a blue color map.

This finding provides evidence that the examination of centers does not reveal any regions or voxels that exhibit statistically significant differences between individuals with ASC and healthy controls. While the NYU center did reveal certain regions with apparent differences, these findings were not replicated across other centers. The lack of replication diminishes the statistical power of the analysis, which, had it been achieved, might have strengthened the evidence for potential differences. Even in such a scenario, however, the results from SPM did not corroborate the findings, highlighting the inconsistency between statistical methods. This discrepancy, combined with the limited sample sizes in most centers and the absence of regions demonstrating substantial differences relative to the permutation tests, reinforces the challenges in identifying reliable structural differences in ASC using this dataset.

### 4.3 Comparison With Existing Literature

With the public availability of large databases and the expansion of machine learning research, numerous interdisciplinary autism studies are utilizing artificial intelligence tools to detect subtle neuroimaging differences, often reporting positive outcomes. Many studies have identified differences in brain volume within the ABIDE I database, identifying specific brain regions such as superior frontal gyrus, middle and superior temporal gyrus, precentral gyrus and postcentral lobule(Ali et al. 2022; Gao et al. 2021; Riddle et al. 2017; Subbaraju et al. 2015; Zhang et al. 2018). SAM analysis highlights significant differences in similar brain regions, such as Precentral_L, Precentral_R, Frontal_Sup_R, Temporal_Mid_L, Temporal_Mid_R, and in Table 4, which collects all the PD values, the postcentral region also stands out. Furthermore, after analyzing each center using SAM, it was observed that some centers did not exhibit any significant differences during the 1000 permutations, not even due to randomness. Conversely, there were centers that showed regions with consistently similar levels of significance across the 1000 permutations, illustrated in Figure 4.

**TABLE 4:** Comparison of SAM and SPM analyses for the entire database and the NYU Center, illustrating probability of detection (*P*) values for HC versus ASC comparison and false positive (FP) values for HC versus HC comparison.

ROI	SAM		SPM	
	Database Pd(FP)	NYU Pd(FP)	Database Pd(FP)	NYU Pd(FP)
Precentral_L	0.252 (0.174)	0.117 (0.074)	0 (0.002)	0 (0.002)
Precentral_R	0.184 (0.138)	0.104 (0.070)	0 (0)	0.002 (0)
Frontal_Sup_L	0.140 (0.136)	0.018 (0.018)	0.003 (0.002)	0 (0)
Frontal_Sup_R	0.227 (0.153)	0.026 (0.021)	0 (0)	0 (0)
Frontal_Sup_Orb_L	0.004 (0.004)	0.002 (0.002)	0 (0)	0 (0)
Frontal_Sup_Orb_R	0.003 (0.002)	0.004 (0.003)	0 (0)	0.001 (0)
Frontal_Mid_L	0.217 (0.156)	0.044 (0.031)	0 (0.001)	0.002 (0.001)
Frontal_Mid_R	0.202 (0.143)	0.038 (0.027)	0 (0)	0 (0)
Frontal_Mid_Orb_L	0.140 (0.106)	0.030 (0.020)	0.003 (0.002)	0 (0)
Frontal_Mid_Orb_R	0.054 (0.054)	0.007 (0.006)	0 (0)	0 (0)
Frontal_Inf_Oper_L	0.005 (0.005)	0.003 (0)	0 (0)	0.001 (0)
Frontal_Inf_Oper_R	0.004 (0.003)	0.007 (0.004)	0 (0)	0 (0)
Frontal_Inf_Tri_L	0.032 (0.017)	0.014 (0.006)	0.001 (0.001)	0 (0)
Frontal_Inf_Tri_R	0.011 (0.005)	0.002 (0.002)	0 (0)	0 (0)
Frontal_Inf_Orb_L	0.004 (0.003)	0.001 (0.001)	0 (0)	0 (0)
Frontal_Inf_Orb_R	0.008 (0.007)	0.001 (0.001)	0 (0)	0 (0)
Rolandic_Oper_L	0.062 (0.027)	0.040 (0.013)	0.002 (0.001)	0.003 (0.002)
Rolandic_Oper_R	0.061 (0.024)	0.010 (0.010)	0.002 (0.001)	0.003 (0.002)
Supp_Motor_Area_L	0.002 (0.002)	0.000 (0.000)	0 (0)	0 (0)
Supp_Motor_Area_R	0.077 (0.059)	0.099 (0.023)	0.001 (0.001)	0.002 (0)
Olfactory_L	0 (0)	0 (0)	0.001 (0)	0 (0)
Olfactory_R	0 (0)	0 (0)	0 (0)	0 (0)
Frontal_Sup_Medial_L	0.057 (0.059)	0.007 (0.008)	0.002 (0)	0.001 (0.001)
Frontal_Sup_Medial_R	0.032 (0.027)	0.004 (0.002)	0 (0)	0 (0)
Frontal_Mid_Orb_L	0.002 (0)	0.001 (0.001)	0.001 (0)	0 (0)
Frontal_Mid_Orb_R	0 (0.001)	0.002 (0)	0.002 (0)	0 (0)
Rectus_L	0 (0)	0 (0)	0 (0)	0 (0)
Rectus_R	0 (0.001)	0 (0)	0.001 (0)	0.001 (0)
Insula_L	0.008 (0.002)	0.012 (0.004)	0 (0)	0 (0)
Insula_R	0.016 (0.009)	0.008 (0.009)	0.002 (0.001)	0 (0)
Cingulum_Ant_L	0.003 (0.005)	0.004 (0.009)	0 (0)	0.001 (0)
Cingulum_Ant_R	0.002 (0.004)	0.011 (0.009)	0.001 (0)	0 (0)
Cingulum_Mid_L	0.009 (0.041)	0.042 (0.020)	0.003 (0.001)	0 (0)
Cingulum_Mid_R	0.017 (0.029)	0.039 (0.030)	0.005 (0)	0.001 (0)
Cingulum_Post_L	0 (0)	0.006 (0.002)	0 (0)	0 (0.001)
Cingulum_Post_R	0 (0)	0.007 (0.001)	0 (0.001)	0 (0)
Hippocampus_L	0.001 (0)	0.011 (0.004)	0 (0)	0.001(0)
Hippocampus_R	0.002 (0.003)	0.005 (0)	0.002 (0)	0.007 (0)
ParaHippocampal_L	0 (0.005)	0 (0.001)	0.005 (0)	0.001 (0.001)
ParaHippocampal_R	0 (0.010)	0.003 (0.004)	0.005 (0)	0.002 (0.001)
Amygdala_L	0 (0)	0 (0)	0.001 (0)	0.003 (0)
Amygdala_R	0 (0)	0 (0.001)	0 (0)	0 (0)
Calcarine_L	0.095 (0.129)	0.118 (0.021)	0.001 (0)	0.001 (0.001)
Calcarine_R	0.062 (0.220)	0.213 (0.084)	0.002 (0)	0 (0.002)
Cuneus_L	0.054 (0.056)	0.007 (0.014)	0.002 (0.001)	0 (0.001)
Cuneus_R	0.126 (0.220)	0.191 (0.118)	0.002 (0)	0.001 (0)
Lingual_L	0.126 (0.202)	0.232 (0.047)	0.001 (0)	0.001 (0.001)
Lingual_R	0.136 (0.204)	0.191 (0.071)	0.002 (0)	0 (0)
Occipital_Sup_L	0.047 (0.053)	0.018 (0.037)	0.001 (0)	0 (0)
Occipital_Sup_R	0.089 (0.080)	0.068 (0.056)	0 (0)	0 (0)
Occipital_Mid_L	0.281 (0.207)	0.124 (0.085)	0.001 (0)	0 (0)
Occipital_Mid_R	0.227 (0.167)	0.117 (0.069)	0.001 (0)	0 (0)
Occipital_Inf_L	0.033 (0.027)	0.029 (0.011)	0 (0)	0 (0)
Occipital_Inf_R	0.048 (0.050)	0.055 (0.029)	0 (0)	0 (0)
Fusiform_L	0.025 (0.076)	0.015 (0.014)	0.001 (0.001)	0.001 (0)
Fusiform_R	0.059 (0.076)	0.052 (0.014)	0.001 (0)	0 (0.001)
Postcentral_L	0.231 (0.197)	0.083 (0.056)	0 (0)	0 (0)
Postcentral_R	0.328 (0.272)	0.131 (0.101)	0.001 (0)	0.002 (0)
Parietal_Sup_L	0.132 (0.066)	0.028 (0.046)	0 (0)	0 (0)
Parietal_Sup_R	0.086 (0.047)	0.010 (0.020)	0 (0)	0.001 (0)
Parietal_Inf_L	0.106 (0.122)	0.052 (0.033)	0.001 (0)	0.001 (0)
Parietal_Inf_R	0.035 (0.011)	0.007 (0.003)	0.001 (0)	0 (0)
SupraMarginal_L	0.022 (0.007)	0.005 (0)	0 (0)	0 (0)
SupraMarginal_R	0.108 (0.049)	0.008 (0.014)	0 (0.001)	0 (0)
Angular_L	0.009 (0.006)	0.006 (0.005)	0 (0.001)	0 (0)
Angular_R	0.092 (0.050)	0.018 (0.014)	0.001 (0)	0 (0)
Precuneus_L	0.140 (0.211)	0.045 (0.050)	0 (0)	0 (0.001)
Precuneus_R	0.114 (0.151)	0.117 (0.060)	0.005 (0.001)	0 (0)
Paracentral_Lobule_L	0.025 (0.041)	0.082 (0.057)	0 (0)	0 (0)
Paracentral_Lobule_R	0 (0.003)	0.033 (0.010)	0 (0)	0 (0)
Caudate_L	0.001 (0.001)	0.004 (0.001)	0 (0)	0 (0)
Caudate_R	0.001 (0)	0.001 (0.001)	0.001 (0)	0 (0)
Putamen_L	0 (0)	0 (0)	0.014 (0)	0.002 (0)
Putamen_R	0 (0.002)	0 (0)	0.016 (0)	0.001 (0.001)
Pallidum_L	0 (0)	0 (0)	0.004 (0)	0.003 (0)
Pallidum_R	0 (0)	0 (0)	0.006 (0)	0.002 (0)
Thalamus_L	0 (0)	0 (0)	0.002 (0)	0.017 (0)
Thalamus_R	0 (0)	0 (0)	0.01 (0)	0.018 (0)
Heschl_L	0 (0)	0.002 (0.001)	0 (0)	0 (0)
Heschl_R	0 (0)	0.004 (0)	0.002 (0)	0 (0)
Temporal_Sup_L	0.032 (0.057)	0.051 (0.018)	0.002 (0)	0.001 (0)
Temporal_Sup_R	0.040 (0.047)	0.023 (0.010)	0 (0.001)	0 (0)
Temporal_Pole_Sup_L	0 (0)	0 (0)	0.001 (0)	0 (0.001)
Temporal_Pole_Sup_R	0.001 (0.003)	0 (0)	0.001 (0)	0 (0)
Temporal_Mid_L	0.151 (0.153)	0.021 (0.022)	0.003 (0.001)	0 (0)
Temporal_Mid_R	0.172 (0.180)	0.037 (0.040)	0.001 (0.001)	0.001 (0)
Temporal_Pole_Mid_L	0 (0)	0 (0)	0 (0)	0 (0)
Temporal_Pole_Mid_R	0.001 (0)	0 (0)	0 (0)	0 (0)
Temporal_Inf_L	0.041 (0.044)	0.001 (0.003)	0.002 (0)	0 (0)
Temporal_Inf_R	0.067 (0.065)	0.006 (0.014)	0 (0)	0 (0.001)
Cerebelum_Crus1_L	0.001 (0.001)	0 (0)	0 (0)	0 (0.001)
Cerebelum_Crus1_R	0.002 (0.001)	0.001 (0.001)	0.001 (0)	0 (0)
Cerebelum_Crus2_L	0.002 (0)	0 (0)	0.001 (0)	0 (0)
Cerebelum_Crus2_R	0.002 (0)	0.001 (0)	0 (0)	0 (0)
Cerebelum_3_L	0 (0)	0.001 (0)	0.001 (0)	0 (0)
Cerebelum_3_R	0.001 (0)	0.005 (0.004)	0 (0)	0 (0)
Cerebelum_4_5_L	0.004 (0)	0.005 (0)	0 (0)	0 (0)
Cerebelum_4_5_R	0.001 (0.001)	0.005 (0)	0.001 (0)	0 (0)
Cerebelum_6_L	0.001 (0.002)	0.005 (0)	0.001 (0.001)	0 (0)
Cerebelum_6_R	0.004 (0.003)	0.012 (0.001)	0.001 (0)	0 (0)
Cerebelum_7b_L	0 (0)	0.001 (0)	0 (0)	0 (0)
Cerebelum_7b_R	0 (0)	0 (0)	0 (0)	0 (0)
Cerebelum_8_L	0.006 (0.007)	0.002 (0)	0 (0)	0 (0)
Cerebelum_8_R	0.009 (0.007)	0.004 (0.001)	0 (0)	0 (0)
Cerebelum_9_L	0.006 (0.006)	0.024 (0.004)	0 (0)	0 (0)
Cerebelum_9_R	0.005 (0.003)	0.020 (0.006)	0 (0)	0 (0)
Cerebelum_10_L	0 (0)	0 (0.001)	0 (0)	0 (0)
Cerebelum_10_R	0 (0)	0 (0)	0 (0)	0 (0)
Vermis_1_2	0 (0)	0 (0)	0 (0)	0 (0)
Vermis_3	0.001 (0)	0.005 (0)	0 (0)	0 (0)
Vermis_4_5	0.001 (0.001)	0.002 (0.002)	0.009 (0)	0.001 (0)
Vermis_6	0 (0)	0 (0)	0 (0)	0 (0)
Vermis_7	0 (0)	0 (0)	0 (0)	0 (0)
Vermis_8	0 (0)	0.001 (0.001)	0.001 (0)	0 (0)
Vermis_9	0 (0)	0.002 (0)	0.001 (0)	0 (0)
Vermis_10	0 (0)	0 (0)	0 (0)	0 (0)

The presence of artifacts and confounding effects in the sample realization provides “actual” classes and subclusters in the population, resulting in heterogeneous samples. Consequently, with a small number of samples, null empirical errors can be reached, compensating for the ultra‐conservative upper bounds, even in low‐dimensional scenarios. Therefore, SAM's methodology only reflects the actual classes that conform to the groups. Although these results may not meet the threshold for statistical significance, they contribute to narrowing down the search for key brain regions that can reliably differentiate between individuals with autism and neurotypical individuals. In contrast, SPM analysis reveals most differences in the central and inner brain regions, consistent with voxel‐wise differences related to registration artifacts or arising from natural anatomical variability between individuals.

Considering previous studies indicating brain differences related to sex within the autism spectrum(Górriz et al. 2019), the differentiation of male subjects from female subjects was studied. Focusing solely on male patients due to their larger representation in the dataset, the results obtained did not deviate from previous findings, suggesting that the sex of the patient did not significantly influence the outcomes, as shown in Figure 9b. This lack of impact could be attributed to the small proportion of female patients in the dataset, limiting their statistical relevance.

The findings of the analysis of the images of the ABIDE I database based on the subjects' condition indicate the absence of any specific brain region that can be considered crucial to distinguish individuals with ASC from healthy controls. The lack of statistically significant differences suggests that a comprehensive differentiation between the two groups cannot be achieved solely through this approach. This finding aligns with previously reported accuracies (<60%) compatible with random classification in ML methods for ASC diagnosis with sMRI of ABIDE database(Katuwal et al. 2015). Attempting to study such a complex disorder using a database that comprises multiple centers with distinct imaging protocols, equipment, and laboratory settings, among other variables, may also yield inconsistent results(Saponaro et al. 2022; Katuwal et al. 2016).

In summary, several confounding factors need to be carefully considered in autism research using the ABIDE database that may obscure the subtle differences in ASC brain structure. These include the existence of sub‐classes within the ASC group(Lombardo et al. 2019), the use of different software solutions in pre‐processing(Katuwal et al. 2016), sex or age differences(Górriz et al. 2019; Katuwal et al. 2015), varying protocols in a multisite database(Saponaro et al. 2022), or even other sources of variability such as asyntomatic incidental findings(Monterrey et al. 2017). The lack of statistically significant differences in regional brain structure suggests a high risk of overfitting when building machine learning models for ASC diagnosis with the structural images of the ABIDE database. Therefore, validation techniques, rigorous estimation of the generalization performance, explicit control of multicenter heterogeneity and the explainability of the results are fundamental to providing insights in autism research with the ABIDE database, not only restricted to the sMRI data but also can be applicable to the fMRI analysis.

## 5 Conclusions

This study aimed to identify specific brain regions in structural MRI that could serve as reliable biomarkers for ASC, utilizing the diverse, multisite ABIDE I database. Using permutation tests alongside SAM and SPM methodologies, we conducted a comprehensive analysis to explore potential structural differences by examining data by center and by condition. In addition, we considered the influence of potential confounding factors, by including noisy images, on the observed results.

Our findings did not reveal any brain region in the center‐by‐center analysis that was consistently replicated between multiple centers, nor did we observe coherence between the results obtained with SAM and SPM. When analyzing differences by condition, we found a similar significance in differences between regions in HC versus HC comparisons and HC versus ASC, suggesting that observed variations were more likely due to factors unrelated to the disorder itself. Importantly, none of the detected differences approached statistical significance.

The main finding of the present analysis suggests that no significant differences can be observed in gray matter tissues of any specific brain region between individuals with ASC and HC subjects in structural MRI images of the ABIDE I database. This finding does not definitively dismiss the possibility of the existence of specific structural differences implicated in autism. However, it does suggest the need for more harmonized datasets and advanced analytical approaches to avoid over‐fitting in ML models and to further investigate the structural foundations of ASC.

## Author Contributions

**F. J. Alcaide**: data curation, visualization, writing –original draft. **I. A. Illan**: formal analysis, investigation, supervision, visualization, writing – original draft, writing –review and editing. **J. Ramírez**: funding acquisition, project administration, supervision, writing – review and editing. **J. M. Gorriz**: conceptualization, data curation, formal analysis, funding acquisition, investigation, methodology, project administration, resources, software, supervision, validation, writing –review and editing.

## Funding

This research is part of the PID2022‐137451OB‐I00 and PID2022‐137629OA‐I00 projects, funded by the MICIU/AEI /10.13039/501100011033 and by FEDER, EU. This work was supported by the Consejería de Economía, Innovación, Ciencia y Empleo, Junta de Andalucía (Grant / Award Number: ‘CV20‐4525') Universidad de Granada (Grant / Award Number: ‘A‐TIC‐080‐UGR18’, ‘B‐TIC‐586‐UGR20’, ‘P20‐00525') .

## Conflicts of Interest

The authors declare no conflicts of interest.

## Figures and Tables

**FIGURE A.1:**
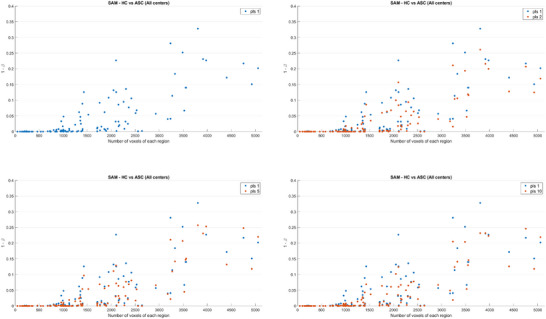
Subplot on comparison between different values of dimensions for Partial Least Squares Regression (PLS) as a method of feature extraction in SAM analysis for all centers HC versus ASC comparison.

**FIGURE A.2:**
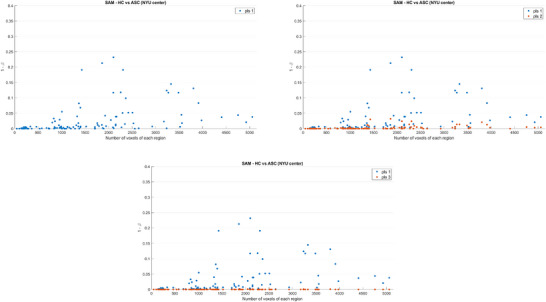
Subplot on comparison between different values of dimensions for PLS as a method of feature extraction in SAM analysis for NYU center HC versus ASC comparison.

## Data Availability

The data that support the findings of this study are openly available in Abide I at https://fcon_1000.projects.nitrc.org/indi/abide/abide_I.html.

## APPENDIX A A

### A.1 Hypothesis Tests

The study involves conducting a hypothesis test, where the null hypothesis ($H_{0}$) represents the accepted belief thus far, which assumes no brain differences between individuals with autism and those without autism. The alternative hypothesis ($H_{1}$), the contrast to the null hypothesis, suggests the presence of significant differences in the brains of autistic and non‐autistic individuals.

The significance level α is set at the standard value of 0.05, indicating the threshold for determining statistical significance. During the statistical analysis of the brain images, if the probability or p‐value of the obtained results for the different brain regions is found to be less than the predetermined significance level α (< 0.05), it would indicate that the observed findings are highly unlikely to occur by chance alone. In such a case, the null hypothesis would be rejected, and it would be concluded that there are statistically significant brain differences.

### A.2 Selection of PLS Dimension

SAM employs a FES stage on each ROI within the images. In this study, the feature extraction method chosen is PLS, with the dimension set to 1. The selection of 1 as the dimension of PLS is based on the observation that increasing the number of coefficients or dimensions leads to less significant results, as depicted in Figures A.1 and A.2. These figures illustrate the HC vs ASC comparisons conducted on both the entire image dataset according to their condition (Figure A.1) and the individual dataset from the NYU center (Figure A.2). Only this comparison is presented since it is of utmost importance in this study, and the NYU center is highlighted due to its substantial individual sample size.

As the dimension of PLS increases, the frequency values of the brain regions generally decline, particularly in the case of the NYU center where this reduction is more pronounced. This implies that the utilization of a large number of PLS components poses a potential risk of overfitting in heterogeneous environments. This risk arises from the fact that PLS aims to maximize the Fisher discriminant ratio (FDR) between the two groups analyzed. Consequently, as the number of PLS components increases, the selected features become more complex and adapted to the specific characteristics of the data. Therefore, the selection of 1 as the PLS dimension is the most suitable option for the analysis throughout this study.
